# Identifying hearing impairment and the associated impact on the quality of life among the elderly residing in retirement homes in Pretoria, South Africa

**DOI:** 10.4102/sajcd.v68i1.788

**Published:** 2021-03-01

**Authors:** Samantha M. Govender, Marguerite de Jongh

**Affiliations:** 1 Department of Speech-Language Pathology & Audiology, School of Health Sciences, Sefako Makgatho Health Sciences University, Pretoria, South Africa

**Keywords:** hearing impairment, retirement homes, presbycusis, pure tone audiometry (Pta), distortion product otoacoustic emissions (DPOAEs), quality of life

## Abstract

**Background:**

Age-Related Hearing Loss (ARHL) is the most widespread sensory disorder in the elderly. Poor audiological support within retirement homes is one of the fundamental issues impacting the Quality of Life (QoL).

**Objectives:**

The objectives of this study were to: (1) Identify the presence of hearing impairment through a hearing screening test battery in a sample of elderly participants residing in three retirement homes. (2) Determine the psychological, communication-related and social impact of the hearing impairment on the QoL in a sample of elderly participants residing in retirement homes.

**Method:**

A prospective cross-sectional research design with quantitative methods of data analysis was used to obtain data from 70 elderly participants (mean age = 79 years, 79% were female). Ten of them used hearing aids. Hearing screening was conducted using otoscopy, tympanometry and air conduction screening (500 Hz–4 kHz). The World Health Organization Quality of Life (WHOQoL) questionnaire was adapted and utilised for the present study.

**Results:**

Findings revealed that 77% of the sample obtained a refer result from the hearing screening protocol indicating a high presence of hearing impairment. Twenty-nine per cent (*n* = 16) of the participants felt depressed, worried and anxious because of their hearing loss and 20% of participants felt unaccepted by their significant others as a result of their hearing impairment. Hearing difficulties were significantly associated with a reduced QoL (*p* = 0.045). Eight of the 10 participants who wore hearing aids reported an overall improvement in QoL since receiving and utilising their hearing aids and 20% (*n* = 2) of hearing aid users reported challenges with maintaining their hearing aids.

**Conclusion:**

The findings of this study emphasised the need for an increased role for audiological services provided by audiologists within retirement homes, thereby contributing to an improved QoL.

## Introduction and literature review

Hearing impairment, the most wide-spread sensory disorder in the elderly, is of growing concern (Mulwafu, Kuper & Ensink, 2016; Quaranta et al., 2015). Globally; it is estimated that 25% of the geriatric population aged between 65 and 75 years, and 70%–80% over 75 years present with age-related hearing loss (ARHL) with an overall prevalence of approximately 44% in adults 65 years and older in Sub-Saharan Africa (Guthrie et al., 2018; Homans et al., 2017; World Health Organization [WHO], 2004). Age-related hearing loss, also known as Presbycusis is a multifactorial disorder that is associated with a shift in hearing sensitivity because of a degenerating auditory system (D’Haese, Van Rompaey, De Bodt, & Van de Heyning, 2019), environmental factors (Lee, 2013) and/or medical conditions (Quaranta et al., 2015). Because of this shift in hearing sensitivity, the elderly experience difficulties in communication and understanding speech in the presence of background noise as well as with social engagements which are important aspects of everyday life and are associated with psychological well-being (Ciorba, Bianchini, Pelucchi, & Pastore, 2012; Moser, Luxenberger, & Freidl, 2017; Quaranta et al., 2015). The effects of ARHL hearing loss may result in reduced Quality of Life (QoL) that is considered a multidimensional concept (Moser et al., 2017) with the elderly experiencing feelings of depression, anxiety, worry, anger and embarrassment and may also be associated with cognitive decline (Ciorba et al., 2012; D’Haese et al., 2019; Sogebi, Olusanya-Peters, & Oluwapeluni, 2013).

The World Health Organisation (1994) describes QoL as individuals’ perception of how their health, wellness, culture, living conditions and environment affect the psychological or emotional state, social relationships, independence, communication and personal beliefs. For the purposes of this paper, QoL will specifically refer to the psychological or emotional impact of hearing impairment on life as well as its impact on social relationships. Studies indicate that elderly individuals residing in retirement homes (both within a state and within the private context) have a greater prevalence of hearing loss and an associated reduction in QoL (Chalise, 2019; McCreedy, Weinstein, Chodosh, & Blustein, 2018; Mirwan et al., 2016; Moroe & Vazzana, 2019). Salonen and colleagues (2013) have identified three fundamental reasons for this. Firstly, poor audiological care for this population is a concern as audiology services are not routinely conducted within retirement homes. Secondly, elderly patients who have been tested and diagnosed with hearing impairment within these homes are inappropriately managed as they are provided with hearing aids but not provided with associated support because of either poor access to healthcare or because of financial limitations. Finally, those who have been fitted with hearing aids do not comply with using and maintaining them because of poor follow-up as a consequence of a negative attitude towards hearing instruments; negative perceptions related to the appearance of hearing instruments on the ear; internal and external stigma and denial of the hearing impairment. Additional factors contributing to reduced QoL in this population relate to social isolation because of stigma around hearing loss and hearing aid use; lack of support including audiologists monitoring of QoL issues during consultations and follow-up visits and lack of family or spousal assistance (Bainbridge & Ramachandran, 2014; Govender & Paken, 2015; Hickson, Worral, Wilson, Tilse, & Sedderfund, 2005; Homans et al., 2017; Moroe & Vazzana, 2019; Patel & Mckinnon, 2018).

The above perusal of literature confirms four important aspects. Firstly, there is a high prevalence of ARHL; secondly, this hearing impairment can lead to a reduced QoL, especially if there is poor hearing aid support. The role of the audiologist in improving QoL for the elderly with ARHL is understated in literature. Finally, literature also affirms that there is a paucity of studies within the South African context relating to hearing loss and associated QoL issues amongst the elderly.

It is therefore worth investigating these issues within the SA context so that findings emerging from such studies may be assimilated into designing contextually relevant hearing screening programmes and evidence-based intervention for the elderly residing in retirement homes. The purpose of the study was therefore, to determine the presence of hearing impairment and its impact on QoL for the elderly residing within retirement home facilities.

## Research method and design

### Aims and objectives

The objectives of the study were to: (1) Identify the presence of hearing impairment through a hearing screening test battery in a sample of elderly participants residing in three retirement homes. (2) Determine the psychological, communication and social impact of the hearing impairment on the QoL in a sample of elderly participants residing in retirement homes.

### Study design

The study employed a cross-sectional research design that aimed to investigate both the presence of hearing impairment through a hearing screening test battery as well as to determine the impact of hearing impairment on QoL through a questionnaire at one point in time in a sample of elderly participants residing in retirement homes.

#### Study sites

Several sites in Pretoria, including both government and private retirement homes, were invited to participate; however, the majority of participants were from private retirement homes with only one state retirement home providing permission.

#### Study sample

A total of five retirement homes, four classified as private retirement homes and one as a state facility, consented to participate, with a total population of 500 elderly individuals within these retirement homes. The minimum recommended sample size for the study was calculated to be 218 by applying a 5% margin of error, 95% confidence level and 50% response distribution. Because of time constraints, participant refusal and cognitive disorders, only 30% (*n* = 70) of the estimated sample size was obtained.

#### Sampling method

Participants were conveniently sampled, based on their availability and willingness to participate in the study.

#### Participant description

The participants’ ages ranged from 65 to 98 years, with a mean age of 79 years. Seventy-nine per cent of the participants were female.

### Data-collection tools

For the hearing screening component of the study, an otoscope, pure-tone audiometer screener (GSI 18), Titan middle ear analyser and DPOAE screener and RS PRO RS-95 Sound Level Meter were calibrated and used for data collection. A questionnaire was utilised to understand the perceived impact of hearing loss on QoL The World Health Organization Quality of Life (WHOQoL) disability questionnaire consists of four domains: namely physical, psychological, social and environmental. The questions relating to social and psychological factors and communication were adapted from the WHOQoL disability questionnaire (WHO, 1998). Additional questions relating to communication challenges and hearing aid use were added. The formulation of the additional questions was guided by perusing literature (Ciorba et al., 2012; Salonen et al., 2013) (see Appendix 1).

### Data-collection procedure

A hearing screening test battery and administration of the questionnaire was conducted on 60 participants and 10 additional participants only completed the QoL questionnaire as they were already diagnosed with hearing impairment and wore a hearing aid.

#### Screening process

**FIGURE 1:**
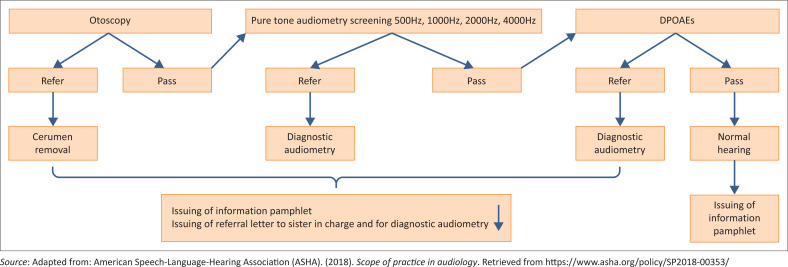
ASHA (2018) screening protocol and pass or refer criteria.

Hearing screening was conducted on all participants (*n* = 60) without hearing aids. Otoscopy, pure tone audiometry (Pta), tympanometry and otoacoustic missions (OAEs) screening were conducted. Pure tone audiometry was conducted over the range of 500 Hz–4 kHz according to the protocol. The intensity level was set at 25 dBHL (ASHA, 2018). Otoacoustic emissions were included because of their sensitivity in detecting cochlear dysfunction, which is associated with ageing. The screening protocol, pass or refer criteria (ASHA, 2018), as well as the referral pathway, are illustrated in Figure 1. All participants received an informational pamphlet regarding ear care at the end of the screening process. Participants who obtained a ‘refer result’ (failed two or more frequencies) were appropriately referred to the relevant professionals such as general practitioners for cerumen management and audiologists for diagnostic testing.

### Questionnaire administration

Participants who underwent hearing screening (*n* = 60) as well as those already fitted with hearing aids (*n* = 10) completed the adapted WHOQoL questionnaire. Because of dexterity and visual problems, the researchers and nursing staff assisted participants in completing the questionnaire by reading out the questions. The researchers conversed in English and Tswana, and for participants that spoke Afrikaans only, the nursing staff were available to translate the questions. The questionnaire comprised simple and easy to follow questions. All of the questions were either yes or no or multiple-choice questions. Participants who used or were fitted with hearing aids previously (*n* = 10) were required to complete an additional section of the questionnaire (Section C), that explored hearing aid use. This section required the participants to mainly respond to yes or no questions and to state their primary challenges with hearing aid use if applicable.

### Data analysis

The data were analysed descriptively through percentage counts and mean averages, and then displayed on graphs and/or pie charts. The Chi-Square test was used to determine the significance between the relationships of variables (presence of hearing impairment and QoL).

### Reliability, validity and bias

The reliability and content validity of the questionnaire utilised in this study was ensured by using questions from a valid and reliable standardised questionnaire (WHOQOL questionnaire, 1998) as well as formulating questions from peer-reviewed articles (Bainbridge & Ramachuandran, 2014; Ciorba et al., 2012). Content validity was additionally ensured as two qualified audiologists scrutinised and evaluated the clarity and relevance of the questions and that the questions met with the objectives of the study. The recommendations made by these audiologists were implemented.

A pilot study was conducted on five participants at one retirement home that was not included in the main study. These five participants were not included in the final analysis of data. Three of these participants were hearing-aid users. The purpose of the pilot study was twofold: (1) to evaluate the reliability of the hearing screening test battery, (2) to test the reliability of the questionnaire. The researchers ensured that the hearing screening equipment was calibrated and therefore, reliable results were obtained. The screening test battery was conducted by researchers in an environment with low ambient noise to improve reliability. The participants were able to respond appropriately to the tonal stimuli, indicating that they understood the instructions. Feedback was given to the participants, referring the participants who failed the screening and obtained a refer result to an audiologist for a diagnostic assessment. Noise levels were noted to be a problem during the pilot study and impacted on the screening test. For the main study, a quiet room was identified, and noise level measurements were routinely taken to monitor noise levels.

The participants needed to verbally respond ‘yes’ or ‘no’ to questions relating to the ease of use of the questionnaire. The questions related to the number of questions, language simplicity and ease of understanding (comprehension). All five participants reported no problems filling in the questionnaire; therefore, no changes were made to the questionnaire.

Bias was likewise avoided through careful design of the data-collection tool; addressing the participants regarding the purpose of the study; the use of simple and accessible language; avoiding of double-barreled questions and testing data-collection tools through a pilot study (Bless, Higson-Smith, & Sithole, 2013). Furthermore, the researchers did not influence any of the responses provided by the participants and analysis bias was avoided by ensuring that the collection and interpretation of the questions were solely completed by writing down the exact participant responses (word-for-word) and selecting a statistician for the data analysis with no ties to the current research study.

### Ethical consideration

Ethical approval to conduct the study was obtained from the School of Health Care Sciences (SMUREC/H/121/2016:U). Furthermore, no participants were harmed or exposed to any risk and confidentiality was assured. Participants willingly provided informed consent and were free to withdraw at any stage. Participants with cognitive challenges were excluded from participation. Confidentiality and anonymity were maintained throughout the study as information derived from this study was not disclosed, and research codes replaced names. Questionnaires and hearing screening results were additionally stored in a locked cabinet and access to the research data was available only to the researchers, research supervisor and the statistician. All participants who obtained a refer result were appropriately referred. Researchers also used calibrated equipment and maintained measures to control infection at all times. Regarding beneficence, all participants received a pamphlet on ear care; participants screened or who required hearing aid support, were appropriately referred.

## Results

The results are discussed in accordance with the objectives of the study.

### Presence of hearing impairment

A total of 60 participants underwent a hearing screening test. Seventy-nine per cent of the participants were female. The racial distribution of the sample size consisted of blacks and whites, with the latter group comprising the largest percentage 91% (*n* = 64). The results obtained from the hearing screening test battery revealed that 23% (*n* = 14) of participants passed the screening and 77% (*n* = 46) of participants obtained a refer result. Of those participants that obtained a ‘*refer*’ result, 15% (*n* = 7) failed the otoscopic examination because of impacted cerumen, 13% (*n* = 6) of participants failed pure tone audiometry (Pta) screening across two or more frequencies and 72% (*n* = 33) failed both Pta and Distortion Product OAEs screening. Sixty-five per cent of participants (*n* = 39) were referred for diagnostic audiometry. Fifteen per cent (*n* = 7) were referred to the general practitioner for cerumen management.

### Impact of hearing impairment on the quality of life

The 60 participants who underwent hearing screening were asked if they perceived themselves as having a hearing problem and of these 51% (*n* = 31) provided an affirmative response. Of the total sample, both those with and without hearing aids (*n* = 70), 74% (*n* = 36) reported that the hearing problem negatively affects their lives. Results revealed that 29% (*n* = 16) of the participants felt depressed, worried and anxious as a result of their hearing difficulties. This was followed by a total of 11% (*n* = 8) of the participants who reported feeling angry about their hearing difficulties. A total of 10% (*n* = 7) felt a sense of grief because of their loss of hearing. Twenty per cent of participants felt unaccepted by their significant others as a result of their hearing impairment.

Regarding social interaction, a total of 78% (*n* = 55) of participants stated yes to the question relating to social participation in that they continue to partake in social gatherings and meetings held at homes and religious gatherings. However, 29% of participants (*n* = 20) reported that their communication partners do get angry and frustrated with them as a result of the hearing problem. In terms of localisation, 66% (*n* = 46) reported that they were able to identify where sounds originated from in the environment, and 33% (*n* = 36) reported challenges with speech discrimination over the phone. A Chi Square test was conducted to determine the relationship between the screening result (*n* = 60) and QoL responses. Hearing difficulties were significantly associated with a reduced QoL (*p* = 0.045, *p* < ?). Of the 60 participants who were screened, 63% (*n* = 38) stated that they had not heard of an audiologist before.

### Hearing aid use and quality of life

All 10 participants who wore hearing aids were fitted with digital hearing aids. Eight of the 10 (80%) participants reported that they used their hearing aids daily with two out of the 10 (20%) saying that they use them infrequently. All participants (*n* = 10) cited challenges with dexterity issues, acoustic feedback and discomfort as reasons for disuse of the hearing aid. Ninety per cent (*n* = 9) of the participants said that since using their hearing aids, their communication abilities improved in terms of sound localisation and listening abilities in the presence of background noise. Six (60%) of the participants mentioned an improvement in localisation to sound since receiving their hearing aids. Eight of the 10 participants further reported an overall improvement in QoL since receiving and utilising their hearing aids, and two of the 10 participants reported challenges with maintaining their hearing aids in terms of changing the batteries and with cleaning their device.

## Implications and recommendations

The results of this study identified possible hearing impairment in more than half of the study sample, requiring further evaluation towards diagnosis and intervention. The study findings correlated with other published studies, relating to the presence of hearing impairment in the elderly (Homans et al, 2017; McCreedy et al., 2018). Another salient finding of the present study was that 15% of participants failed the otoscopic examination because of cerumen impaction. This finding was consistent with other studies (Aremu, Alabi, Segun-Busari, & Ogah, 2010; Sugiura et al., 2014). Reasons cited for the increased presence of impacted cerumen amongst the elderly include thinning of the surface epithelium, atrophy of the subcutaneous tissue, less production of oil and sweat by the ceruminous and sebaceous glands and lengthening of the hair in the ear canal all associated with the ageing of the skin in the ear canal (Sogebi et al., 2013). A large percentage of participants also failed the OAE screening, implying that the cochlear outer hair cells (OHCs) have been affected. This is consistent with the disease progression of ARHL (Fischer, Johnson Chacko, Glueckert, & Schrott-Fischer, 2020).

The current study findings illustrated a statistically significant relationship between the presence of possible hearing impairment (through a screening test battery) and self-reported reduction in QoL. Joore and colleagues (2002) demonstrated that new hearing aid users experienced less anxiety and depression following hearing aid use. Mulrow et al. (1990) also reported a reduction in depression among hearing aid users, as measured by a geriatric depression scale. Participants expressed feelings of anger, grief, frustration and sadness as a result of their difficulties with hearing. In addition, the sample that were fitted with hearing aids, reported improved social and communication interaction because of hearing aid use. However, the hearing aid fitted participants reported problems with managing their devices. These findings are in agreement with the existing body of literature (Ciorba et al., 2012; Nordvik et al., 2018). Chew and Yeak (2010) emphasise that untreated or poorly managed hearing loss can result in significant reduction in QoL in the elderly. The majority of the study sample were either not diagnosed or not adequately managed for hearing impairment and this decline in hearing was associated with their feelings of anger, sadness, worry and depression. Ciorba et al. (2012) hence emphasise the importance of routine evaluation of QoL status during routine audiological management.

Ciorba and colleagues (2012) as well as Tognola, Mainardi, Vincenti and Cuda (2019) found that hearing aid fittings and continued follow-up improve QoL in the elderly. The present study findings aligned to the above assertion in that the participants who used hearing aids reported improved social interaction and communication. A small proportion of the sample of hearing aid users did report challenges in maintaining their hearing aid. It is plausible that the lack of adequate audiological support within the context could be the attributable cause; however, this would require a scientific inquiry to support this assumption. Hearing aid support would improve QoL for the elderly as Said (2017) states that such support for the elderly can result in improved psychosocial and cognitive functioning.

Overall, the results of the present study corroborate with those of Salonen and colleagues (2013), where the three fundamental problems of poor audiological care, poor continuity of care for those diagnosed with hearing loss and poor hearing aid maintenance, were identified as the salient issues relating to audiological care in old age homes. Geriatric hearing screening programmes within retirement facilities and at the community level need to be developed (Solheim, Shiryaeva, & Kvaerner, 2016). However, there is a dearth of such promotion and prevention programmes in the South African context, despite its relevance within the primary healthcare approach. The findings of this study, therefore, place impetus on the development and initiation of hearing screening and (re) habilitative programmes and support within retirement homes and for the elderly in general so that adequate intervention may be provided which could, therefore, mitigate the impact of auditory problems on the QoL. Thus it becomes important that audiology intervention programmes consider the multifaceted nature of ARHL and address not only the hearing difficulties but the associated impact on social, communication and psychological well-being.

## Conclusion

A high prevalence of undiagnosed and poorly managed hearing impairment in the elderly residing in retirement homes is a significant problem. Hearing impairment has an impact on the social, psychological and communication aspects thereby reducing QoL. Audiologists need to solidify their roles and responsibilities for the management of ARHL and ensure that the vulnerable ageing population is adequately provided with the knowledge, resources and services to effectively manage their hearing disorders to reduce the negative impact that ARHL can have on QoL.

Future research is required on the development and feasibility of geriatric hearing screening programmes, particularly for use within retirement home facilities. These programmes should ideally be multidisciplinary, offering evaluation and monitoring of QoL, practical coping mechanisms and psychosocial support, alongside audiological management in order to remediate the progression of the disorder and subsequently impact on the QoL of the elderly in these facilities. Further research into the outcomes of a collaborative approach of different professionals in the management of hearing loss in retirement homes would be warranted. This is important as the progression of presbycusis cannot be prevented, therefore, early identification and subsequent management of the hearing impairment is essential (Ciobra, 2012). Limitations of the study included the small sample size that hindered the generalisation of findings. Furthermore, a comparison of services and QoL outcomes within the private and state homes could not be conducted for this study because of the uneven distribution between both contexts. In addition, more qualitative studies looking into experiences of the current population involving hearing and their QoL as well as interviewing professionals to also assess reasons behind poor or neglectful care, including lack of follow-up, is required.

## Appendix 1

### Questionnaire

**RESEARCH QUESTIONNAIRE: HEARING AIDS USERS**

**IDENTIFYING AUDITORY PATHOLOGY AND ASSOCIATED FACTORS AMONGST THE ELDERLY IN RETIREMENT HOMES IN PRETORIA NORTH.**

**Name of home: __________________________________**

**Research participant number: ______________________**

Thank you for taking the time to participate in the research study. The aim of the study is to identify hearing impairment and to determine factors that affect quality of life amongst the elderly participants residing in retirement homes. We request you to fill in or provide answers to the questions in the questionnaire. All responses will be kept strictly confidential.

### Section A: Biographical information

#### Participant’s Number

**Table:**

Age:	
Gender:	Male : _________________/ Female________________
Contact no:	
Alternative contact no:	

### Section B:

#### Psychological domain

1.1. Do you believe you have a hearing problem?
  Yes       No  
1.2. If you believe you that you have a hearing problem, do you feel that it negatively impacts your life?
  Yes       No  
2. What are the feelings that you experience about your hearing loss? (Tick all that apply.)
  **Table:**
  Worry	
  Sadness	
  Anxiety	
  Depression	
  Blue mood	
  Anger	
  Despair	
  Grief	
3. Do you feel that other people accept you with your hearing loss?
  Yes       No  

#### Social and communication domains

4. Do you engage in social activities such as religious gatherings or meetings held at your retirement home?
  Yes       No  
5. Are you satisfied with your ability to communicate with other people?
  Yes       No  
6. Do people get angry with you when they try to communicate with you?
  Yes       No  
7. Can you localise sounds (identify where sounds come from) in your environment?
  Yes       No  
8. Do you experience difficulty discriminating speech when using the phone?
  Yes       No  
9. Do you feel that other people respect you?
  Yes       No  
10. Do you know who an Audiologist is?
  Yes       No  

### Section C:

#### Complete this section only if you wear a hearing aid

11. Do you have a hearing aid?
  Yes       No  
12. Do you wear your hearing aid? (Tick the answer that applies.)
  **Table:**
  Daily:	
  Now and then (infrequently):	
  Never:	
13. Has your ability to localise sound improved with using the hearing aid?
  Yes       No  
14. Have your communication abilities improved with using a hearing aid?
  Yes       No  

THANK YOU FOR TAKING THE TIME TO COMPLETE THE QUESTIONS!
